# Abdominal aortic stiffness as a marker of atherosclerosis in childhood-onset asthma: a case–control study

**DOI:** 10.5830/CVJA-2014-046

**Published:** 2015

**Authors:** Zülal Ülger, Figen Gülen, Ruhi Arif Özyürek

**Affiliations:** Ege University Children’s Hospital, İzmir, Bornova, Turkey; Ege University Children’s Hospital, İzmir, Bornova, Turkey; Ege University Children’s Hospital, İzmir, Bornova, Turkey

**Keywords:** asthma, children, aortic stiffness

## Abstract

**Background:**

Asthma is one of the chronic inflammatory diseases. It is known that chronic inflammation accelerates atherosclerosis. Abdominal aortic stiffness parameters can be used to detect the early development of atherosclerosis.

**Aim:**

In this study, we aimed to evaluate abdominal aortic stiffness parameters in childhood-onset asthma compared with a control group.

**Methods:**

In this cross-sectional, case–control study, we evaluated 50 patients with childhood-onset asthma, and 57 healthy children as controls. Patients with a diagnosis of asthma of at least three years’ duration were included in the study. Children with hypertension, hyperlipidaemia, diabetes, a history of smoking contact, or systemic disease were excluded. The study and control groups were evaluated with transthoracic echocardiography, and abdominal aorta diameters were measured. Using the measured data, abdominal aortic stiffness parameters (aortic distensibility: DIS, aortic strain: S, pressure strain elastic modulus: Ep, and pressure strain normalised by diastolic pressure: Ep*) were calculated. Statistical evaluation was done with the Student’s *t*-test, chisquared test and Pearson’s correlation test.

**Results:**

The study group consisted of 50 children (24 female, 26 male) with asthma. According to the GINA guidelines, 26 of the patients had mild intermittant asthma, six had mild persistent asthma and 18 had intermediate persistent asthma. None of the patients had severe asthma. In 37 of the asthma patients, spIgE was positive and these patients were accepted as having atopic asthma; 27 of these patients received immunotherapy. We did not detect any differences between the study and control groups in terms of gender, age and body mass index. No differences were evident between the groups with regard to systolic and diastolic blood pressure, heart rate, blood cholesterol levels and respiratory function test parameters. There was no difference between the asthma and control groups in the measurement of abdominal aortic stiffness parameters. There was no significant correlation between aortic stiffness parameters and high-sensitivity C-reactive protein, blood total cholesterol, LDL cholesterol and HDL cholesterol levels.

**Conclusion:**

We did not find any difference between the asthma patients and control group with regard to aortic stiffness parameters (DIS, S, Ep and Ep*) and there was no difference in these parameters when we compared patients with mild asthma with those with moderate asthma. These results may be due to the anti-inflamatory effect of inhaled steroids. Further studies are needed to validate these results.

## Abstract

Asthma is an important health problem in children. Substantial evidence has demonstrated that asthma is a chronic inflammatory disease with activation of the inflammatory cells within the airways. Recent studies have reported that systemic inflammation is related to disease progression in asthma.^1^ The pro-inflammatory cytokines such as tumour necrosis factor alpha (TNFα), interleukin 6 (IL-6) and C-reactive protein (CRP) are elevated in patients with asthma.^1^-^3^

Atherosclerosis and asthma are both chronic inflammatory conditions. Inflammation leads to impairment of endothelial cell function, and chronic inflammation accelarates atherosclerosis.^4^ Elevated arterial stiffness, a marker of subclinical atherosclerosis, is associated with myocardial infarction, heart failure, stroke, renal disease and elevated total mortality rates.^5^

Much research has revealed that patients with asthma are at increased risk of pulmonary embolism, hypertension, coronary heart disease and heart failure.^6^-^9^ Reduction in arterial distensibility leads to increased pulse pressure, and impedance of arterial flow and pulsatile cardiac work load. Arterial stiffness is a mechanical property related to vascular impedance and the afterload that is presented to the left ventricle. Abdominal aortic stiffness increases with age, and in many studies, its usefulness has been demonstrated.^10^-^15^

In the literaure, changes in abdominal aortic stiffness in childhood-onset asthma have not been clearly determined. The purpose of our study was to evaluate abdominal aortic stiffness in patients with childhood-onset asthma.

## Methods

Our study was a cross-sectional, case–control study. Fifty asthma patients (24 girls, 26 boys) aged eight to 17 years, who were followed by the paediatric allergy department of our hospital for at least three years, were included in this study. Children with hypertension, hyperlipidaemia, diabetes, a history of smoking contact and systemic disease were excluded. The asthma diagnosis was established from a history of intermittent wheezing, the presence of reversible airway obstruction and at least 12% improvement in forced expiratory volume in one second (FEV1) following bronchodilator administration.

The Global Initiative for Asthma guidelines (GINA) was used to determine clinical severity of the asthma.^16^ Twenty-six patients had mild intermittant asthma, six had mild persistent and 18 had moderate persistent asthma. Allergen sensitivity in the asthma patients was determined with specific IgE (sIgE) and skin-prick tests to aero-allergens.

sIgE levels were determined with the CAP FEIA method (Pharmacia, Uppsala, Sweden), which detects sensitisation in the serum against inhaled allergens (wild grass, house dust mite, animal dander, yeasts, grass pollen, trees). The result was considered positive if the measured value was greater than 0.35 kU/l.

The skin-prick test (SPT) was done using Allergopharma (Joachim Ganzer KG, Reinbeck, Germany) commercial allergen solutions. A total of 44 different allergens consisting of house dust mite, grass, wild grass, tree pollens, fungi, animal dander and insects were tested and children with at least one positive test were considered atopic. Asthma patients who had a positive sIgE and sensitivity against at least one aero-allergen on the SPT were included in the atopic asthma group.

Immunocompromised patients, patients with a history of chronic inflammation/rheumatological disorders, diabetes, hypertension, hypercholesterolaemia and those with autoimmune diseases or a history of smoking exposure were excluded. Asthma patients with an exacerbation of their asthma within the previous month or with symptoms of respiratory tract infection were also excluded.

The control group consisted of 57 gender- and age-matched healthy children. They were chosen from children referred to the paediatric cardiology out-patient clinics due to innocent murmur. The control group was evaluated with regard to familial and personal history of hyperlipidaemia and atopy, chronic and/or severe infections, and rheumatological and autoimmune diseases. Children were included in the control group if they had no sign of atopic diseases and no personal familial history of atopy. The control group was also selected from non-smoking households.

The local ethics committee approved the study. Informed consent was obtained from the parents of all subjects in the study and control groups.

The patients in the study group and the healthy controls were weighed with an electronic digital scale that was sensitive to 0.1 kg. Body height was measured and body mass index (BMI) was calculated with the formula: weight (kg)/height^2^ (m^2^).

A detailed medical history was obtained and a physical examination was performed by the same paediatric cardiologist. Blood pressure was recorded and all subjects were evaluated with a respiratory function test.

Plasma lipid levels were measured after 12 hours of fasting. Serum total cholesterol, high-density lipoprotein (HDL) and low-density lipoprotein (LDL) cholesterol levels were measured with Alcyon 300 (Abbott Laboratories, USA) equipment by enzymatic methods. High-sensitivity C-reactive protein (hs-CRP) levels in the study and control groups were measured on an automatic analyser, based on the turbidimetry method.

Blood pressure measurements were done after 15 minutes of rest; the right brachial artery pressure was measured by sphygmomanometer with an appropriate cuff. Both systolic (Ps) and diastolic blood pressure (Pd) were measured, and after three measurements the mean value was obtained. Pulse pressure (PP) was calculated as PP = Ps – Pd.

All the patients and control group underwent two-dimensional, M-mode and Doppler studies using GE Vingmed Vivid 7-model echocardiography (GE Vingmed, Ultrasound AS, Horten, Norway) with a 3-MHz transducer. All the subjects were at rest and lying in the left decubitus position during the examination.

End-diastolic left ventricular posterior wall thickness (LVPWTed), left ventricular end-diastolic and systolic diameters (LVED, LVES), left atrial diameter (LA) and aortic anulus diameters were measured. The ejection fraction (EF) and fractional shortening (FS) were measured from M-mode echocardiographic tracings. The measurements were determined with standard techniques in accordance with the recommendations of the American Society of Echocardiography.^17^ Mean pulmonary artery pressure of all subjects was calculated from pulmonary artery accelaration time.

A long-axis view of the abdominal aorta of the subxiphoid area was recorded and maximum systolic (Ds) and minimum diastolic diameter (Dd) was measured by M-mode echocardiography. All echocardiographic measurements were done by the same experienced paediatric cardiologist and intra-observer variability was evaluated with intraclass correlation coefficient (ICC); ICC was 0.9 (excellent reliability).

All aortic measurements were made as previously described by Lacombe *et al.*^10^ Aortic strain (S) was calculated from the changes in aortic diameter, and pressure strain elastic module was also calculated from the aortic strain and the changes in brachial artery systolic and diastolic pressure using the formulae: S = (Ds – Dd)/Dd and Ep = (Ps – Pd)/S.

Pressure strain normalised (Ep*) by diastolic pressure was calculated with the equation: Ep* = Ep/Pd. Aortic distensibility (DIS) was calculated according to the previously proposed and evaluated equations^10^-^15^ as: DIS = [2(Ds – Dd)/Dd(Ps – Pd)] × 10^-6^ cm/dyne.

S and DIS represent the distensibility or elasticity of the aortic wall; Ep and Ep* represent the stiffness of the aortic wall, and Ep and Ep* are the mean stiffness of the aorta. S and Ep* are dimensionless ratios, whereas Ep has a dimension and is represented with the unit of N/m^2^ (force/unit area).

## Statistical analyses

All statistical analyses were performed using Systat statistical software (version 15.0 for Windows; SPSS Inc, Chicago, IL, USA). Data were tested for homogeneity of variance with the Shapiro–Wilk test. The Student’s *t*-test (unpaired) and chi-squared test were used for comparison of statistical difference between the groups. Correlations with the aortic elasticity parameters were evaluated with Pearson’s correlation test. Statistical significance was taken as *p* < 0.05. All data were presented as mean ± SD.

## Results

The study group consisted of 50 children (24 female, 26 male) with asthma. According to the GINA guidlines, 26 of the patients had mild intermittant asthma, six had mild persistent and 18 had intermediate persistent asthma. None of the patients had severe asthma. In 37 of the asthma patients, sIgE was positive and these patients were accepted as the atopic asthma group; 27 of these patients received immunotherapy.

The mean age of the asthma group was 11.7 ± 2.7 years and of the control group, 12.3 ± 2.8 years (34 female, 23 male). There was no difference between the groups in terms of age, gender and BMI (Table 1). No differences were evident between the groups in terms of systolic/diastolic blood pressure, heart rate, blood cholesterol levels and respiratory function test parameters (Table 1).

**Table 1:** Characteristics of the asthma patients and control group

	*Asthma patients (n = 50)*	*Control group (n = 57)*	*p-value*
Gender, female/male	24/26	34/23	> 0.05
Age, years	11.7 ± 2.7	12.3 ± 2.8	> 0.05
Presence of atopy, %	37 (74)	0 (0)	
Immunotherapy	27 (54)	0 (0)	
Duration of diagnosis of asthma, years	8.1 ± 2.8 (3–15)		
Weight, kg	43.0 ± 15.5	47.8 ± 17.0	> 0.05
Height, cm	148.0 ± 15.9	150.4 ± 16.1	> 0.05
BMI, kg/m²	19.0 ± 4.1	20.5 ± 4.3	> 0.05
Systolic blood pressure, mmHg	101.1 ± 10.4	102.4 ± 10.4	> 0.05
Diastolic blood pressure, mmHg	63.5 ± 9.9	64.9 ± 9.8	> 0.05
Mean blood pressure, mmHg	76.0 ± 9.2	77.4 ± 9.3	> 0.05
Heart rate, beat/min	84 ± 15	85 ± 14	> 0.05
Total cholesterol, mg/dl (mmol/l)	152.5 ± 32.6 (3.96 ± 0.84)	147.5 ± 24.6 (3.82 ± 0.64)	> 0.05
LDL cholesterol, mg/dl (mmol/l)	83.6 ± 17.8 (2.17 ± 0.46)	79.6 ± 18.1 (2.06 ± 0.47)	> 0.05
HDL cholesterol, mg/dl (mmol/l)	55.6 ± 17.3 (1.44 ± 0.45)	50.7 ± 11.5 (1.31 ± 0.30)	> 0.05
hs-CRP, mg/dl	2.12 ± 0.41	0.79 ± 0.20	< 0.05
FVC, % predicted	87.3 ± 13.6	87.1 ± 10.6	> 0.05
FEV1, % predicted	97.7 ± 14.9	99.2 ± 11.1	> 0.05
FEV1/FVC, %	95.2 ± 5.8	97.0 ± 4.3	> 0.05
PEF, % predicted	92.8 ± 16.6	89.8 ± 14.6	> 0.05

Data are presented as mean ± standard deviation. BMI: body mass index, FEV1: forced expiratory volume in one second, FVC: forced vital capacity, HDL: high-density lipoprotein, LDL: low-density lipoprotein, PEF: peak expiratory flow.

Asthma patients and the control group were evaluated with transthoracic echocardiography. There was no significant difference between the two groups in the measurements of LVPWTed, LVED, LVES, IVSed, LA, aortic anulus diameter, EF and FS (Table 2). Mean pulmonary pressure (mPAP) of the asthma patients was higher than in the control group (19.9 ± 7.1 vs 12.6 ± 6.2 mmHg) and this difference was statistically significant (*p* < 0.05). There was no correlation between mPAP and aortic stiffness parameters (Pearson’s correlation analysis).

**Table 2:** Echocardiographic findings of the asthma and control groups

	*Asthma patients (n = 50)*	*Control group (n = 57)*	*p-value*
LVPWTed, mm	7.1 ± 0.1	7.0 ± 0.1	> 0.05
LVED, mm	40.1 ± 4.6	40.4 ± 4.8	> 0.05
LVES, mm	25.8 ± 4.5	24.7 ± 3.9	> 0.05
IVSed, mm	7.4 ± 1.1	7.2 ± 1.1	> 0.05
LA, mm	25.3 ± 4.0	23.2 ± 3.7	> 0.05
Aortic anulus, mm	16.8 ± 3.2	17.1 ± 2.6	> 0.05
EF, %	72 ± 10	76 ± 8	> 0.05
FS, %	36 ± 7	38 ± 7	> 0.05
mPAP, mmHg	19.9 ± 7.1	12.6 ± 6.2	<0.05

Data are presented as mean ± standard deviation. EF: ejection fraction, FS: fractional shortening, LA: left atrial diameter, LVED: left ventricular end-diastolic diameter, LVES: left ventricular systolic diameter, LVPWTed: end-diastolic left ventricular posterior wall thickness, mPAP: mean pulmonary artery pressure.

In 15 of the asthma patients, echocardiography revealed mild tricuspid regurgitation and right ventricular systolic pressure was calculated from regurgitant flow. Average right ventricular systolic pressure of these 15 patients was 27.2 ± 5.7 mmHg. Since we had evaluated only asthma patients without exacerbation of their asthma within the previous four weeks, there was no difference in baseline respiratory function test parameters between the study and control groups.

There was no significant difference between the asthma and control groups in the measurements of Ds, Dd, DIS, S, Ep and Ep* (Table 3). There was no significant correlation between aortic stiffness parameters and serum total cholesterol (*r* = 0.03), LDL cholesterol (*r* = 0.09), HDL cholesterol (*r* = 0.09) and triglyceride (*r* = 0.134) levels (Pearson correlation analysis). There was no correlation between hs-CRP and aortic stiffness parameters, DIS (*r* = 0.268), Ep (*r* = 0.199), EP* (*r* = 0.150) and S (*r* = –0.230).

**Table 3:** Aortic stiffness parameters in the asthma and control groups

	*Asthma Patients (n = 50)*	*Control Group (n = 57)*	*p-value*
Peak aortic velocity, cm/s	125.6 ± 16.7	123.5 ± 17.9	> 0.05
Ds, mm	11.4 ± 2.0	11.1 ± 1.9	> 0.05
Dd, mm	8.2 ± 1.5	8.2 ± 1.8	> 0.05
DIS, 10^-6^ cm^2^/dyne	1.35 ± 0.52	1.41 ± 0.66	> 0.05
S	0.38 ± 0.11	0.37 ± 0.14	> 0.05
Ep, N/m^2^	107.5 ± 39.0	116.5 ± 55.9	> 0.05
Ep*	1.75 ± 0.73	1.83 ± 0.90	> 0.05

Data are presented as mean ± standard deviation. Dd: abdominal aorta diastolic diameter, DIS: aortic distensibility, Ds: abdominal aorta systolic diameter, Ep: pressure strain elastic modulus, Ep*: pressure strain normalised by diastolic pressure, S: aortic strain.

Out of 50 asthma patients, 18 had intermediate severity asthma. Aortic stiffness parameters were compared between these patients and the control group. There was no statistically significant difference between the two groups (*p* > 0.05) (Table 4).

**Table 4:** Aortic stiffness parameters in patients with intermediate severity asthma and the control group

	*Intermediateseverity asthma patients (n = 18)*	*Control group (n = 57)*	*p-value*
Peak aortic velocity, cm/s	125.6 ± 16.7	123.5 ± 17.9	> 0.05
Ds, mm	11.4 ± 2.0	11.1 ± 1.9	> 0.05
Dd, mm	8.2 ± 1.5	8.2 ± 1.8	> 0.05
DIS, 10^-6^ cm^2^/dyne	1.31 ± 0.51	1.41 ± 0.66	> 0.05
S	0.39 ± 0.10	0.37 ± 0.14	> 0.05
Ep, N/m^2^	105.9 ± 42.4	116.5 ± 55.9	> 0.05
Ep*	1.71 ± 0.74	1.83 ± 0.90	> 0.05

Data are presented as mean ± standard deviation. Dd: abdominal aorta diastolic diameter, DIS: aortic distensibility, Ds: abdominal aorta systolic diameter, Ep: pressure strain elastic modulus, Ep*: pressure strain normalised by diastolic pressure, S: aortic strain.

Twenty-six of the asthma patients were intermittently using inhalers with short-acting beta-agonists; 10 were also using montelukast Na together with short-acting beta-agonists. Twenty-four of the patients were using long-acting inhalers with beta-agonists together with inhalers with corticosteroids; 16 of them were also using montelukast Na. Since these asthma drugs have multiple effects on the aortic and peripheral vascular system, we compared the aortic stiffness parameters of these different treatment groups. We did not detect statistically significant differences between the groups (*p* > 0.05).

We evaluated the effects of the presence of atopy and severity of asthma on aortic stiffness parameters. There was no difference in aortic stiffness parameters between atopic asthma patients and the control group (Table 5).

**Table 5:** Aortic stiffness parameters in asthma patients with atopy and the control group

	*Asthma patients with atopy (n = 37)*	*Control group (n = 57)*	*p-value*
Peak aortic velocity, cm/s	128.0 ± 16.5	123.5 ± 17.9	> 0.05
Ds, mm	11.5 ± 1.9	11.1 ± 1.9	> 0.05
Dd, mm	8.3 ± 1.3	8.2 ± 1.8	> 0.05
DIS, 10^-6^ cm^2^/dyne	1.31 ± 0.51	1.41 ± 0.66	> 0.05
S	0.38 ± 0.12	0.37 ± 0.14	> 0.05
Ep, N/m^2^	105.4 ± 35.6	116.5 ± 55.9	> 0.05
Ep*	1.69 ± 0.66	1.83 ± 0.90	> 0.05

Data are presented as mean ± standard deviation. Dd: abdominal aorta diastolic diameter, DIS: aortic distensibility, Ds: abdominal aorta systolic diameter, Ep: pressure strain elastic modulus, Ep*: pressure strain normalised by diastolic pressure, S: aortic strain.

## Discussion

The present cross-sectional study was undertaken to comparatively evaluate the elastic properties of the abdominal aorta in children with asthma and in a control group. Our hypothesis was that since asthma is a chronic inflammatory disease, it could lead to the early development of atherosclerosis in childhood-onset asthma.

To detect the effect of inflammation, we included patients with a diagnosis of asthma of at least three years’ duration. As a marker of atherosclerosis, we evaluated abdominal aortic stiffness parameters with transthoracic echocardiography. Stiffness and distensibility assessments of the abdominal aorta play an important role in evaluation of the elasticity of the arterial system. If there is atherosclerosis, aortic stiffness, Ep and Ep* will increase, whereas DIS and S will decrease. S and DIS represent the elasticity of the abdominal aortic wall.

In the literature, aortic distensibility has been shown to be useful in adults as a non-invasive method to detect early atherosclerosis. The increased stiffness causes an increase in pulse pressure and a decrease in diastolic blood pressure, thereby causing increased left ventricular afterload and increased fatigue in arterial wall tissues. Previous studies have shown that measurement of aortic stiffness helps in the early detection of atherosclerosis, and the abdominal aorta becomes stiffer with age, hypertension, atherosclerosis, tobacco-smoking, obesity, and in β-thalassaemia patients and patients with Marfan syndrome and Kawasaki disease.^11^,^13^,^14^

Lacombe *et al.* demonstrated that in subjects older than 20 years of age, S, Ep and Ep* were related to age due to atherosclerosis.^10^ We calculated S, Ep, Ep* and distensibility using the formula proposed by Lacombe and Lage *et al.*^10^,^15^ Okubo found that aortic distensibility varies with age; it was low in infants, increased gradually to a peak from 10–15 years, and then decreased with age.^11^

Atherosclerosis and asthma are both chronic inflammatory diseases. Inflammation leads to impairment of endothelial function. When the inflammation is chronic, this causes acceleration of atherosclerosis.^18^ In the literature, some studies have stated that asthma itself could be a risk factor for heart disase and stroke.^19^,^20^

Related to increased oxidative stress, asthma is a chronic inflammatory disease.^21^ The association between chronic inflammation and oxidative stress is well documented. In asthma patients with inflammatory conditions, elevated levels of reactive oxygen species, such as hydroxyl radicals, superoxides and peroxides have been reported.^22^ Chronic inflammation has also been increasingly associated with endothelial dysfunction, atherosclerosis and arterial stiffness, and these in turn with adverse cardiovascular events and common inflammatory pathways.^23^,^24^

Some studies have evaluated the relationship between adult-onset asthma and atherosclerosis but the results are contradictory. Onufrak *et al.* showed that in adult-onset asthma patients, the risk of atherosclerosis was increased.^25^ However, in another study by Otsuki *et al.*, carotid atherosclerosis was reduced in asthmatic adult patients treated with inhaled corticosteroids compared with matched controls, and they found that inhaled corticosteroids had protective effects against atherosclerosis.^26^

Weiler *et al.* found significant correlations between measurements of periferal arterial stiffness and FEV1 in adult asthmatics, and suggested the presence of a common systemic, most likely inflammatory pathway involving both the cardiovascular and respiratory systems.^27^ In another study of adult asthmatics, Sun *et al.* found that that patients with severe asthma had increased brachial–ankle pulse-wave velocity (baPWV) compared with those with stable asthma and control subjects. Furthermore, baPWV was elevated in patients with stable asthma compared with the control subjects.^28^

However, there are only a few studies evaluating the relationship between childhood-onset asthma and atherosclerosis. In the study of Cakmak *et al.*,^29^ carotid intima–media thickness (CIMT) in asthmatic children was found to be higher compared to the control group and there was a positive correlation between CIMT and total oxidant status. They studied only children with mild asthma who were not using prophylactic inhaled corticosteroids.

In all these studies, CIMT was evaluated as a marker of atherosclerosis. However, in our study, we evaluated abdominal aortic stiffness as a sign of atherosclerosis in childhood asthma.

In our investigation, the study population consisted of children with stable asthma. We did not evaluate aortic stiffness parameters in children with severe asthma. Further studies including larger population size and children with severe asthma may reveal different results regarding abdominal aortic stiffness.

In our study, we used only hs-CRP as an inflammatory marker. An inflammatory marker showing oxidant status could not be studied.

## Conclusion

We evaluated aortic stiffness parameters in childhood-onset asthma in children with a diagnosis of asthma of at least three years’ duration (average duration from diagnosis: 8.1 ± 2.8 years). We did not find any difference between chidhood-onset asthma patients and the control group with regard to aortic stiffness parameters (DIS, S, Ep and Ep*). There was also no difference in these parameters when we compared patients with mild asthma and those with moderate asthma. These results may be due to the anti-inflammatory effect of inhaled corticosteroid treatment.

Since there is no study in the literature evaluating abdominal aortic stiffness in childhood-onset asthma patients, we could not compare our results with other studies. Further studies are needed to validate these results.
